# Combining Electronic Tongue Array and Chemometrics for Discriminating the Specific Geographical Origins of Green Tea

**DOI:** 10.1155/2013/350801

**Published:** 2013-07-15

**Authors:** Lu Xu, Si-Min Yan, Zi-Hong Ye, Xian-Shu Fu, Xiao-Ping Yu

**Affiliations:** Zhejiang Provincial Key Laboratory of Biometrology and Inspection & Quarantine, College of Life Sciences, China Jiliang University, Hangzhou 310018, China

## Abstract

The feasibility of electronic tongue and multivariate analysis was investigated for discriminating the specific geographical origins of a Chinese green tea with Protected Designation of Origin (PDO). 155 Longjing tea samples from three subareas were collected and analyzed by an electronic tongue array of 7 sensors. To remove the influence of abnormal measurements and samples, robust principal component analysis (ROBPCA) was used to detect outliers in each class. Partial least squares discriminant analysis (PLSDA) was then used to develop a classification model. The prediction sensitivity/specificity of PLSDA was 1.000/1.000, 1.000/0.967, and 0.950/1.000 for longjing from Xihu, Qiantang, and Yuezhou, respectively. Electronic tongue and chemometrics can provide a rapid and reliable tool for discriminating the specific producing areas of Longjing.

## 1. Introduction

Green tea, unfermented and made from the leaves of the *Camellia sinensis* plant, is one of the most popular beverages consumed across the world [1–3]. The property and chemical components of green teas are influenced by many factors, such as tea species, harvest season, climate, geographical locations, and processing. In China, among various factors, the geographical origin is recognized as an important aspect of tea. Because of the similar tea species, cultivation and processing conditions in a specific tea-producing area, many teas are named after their geographical origins.

Longjing tea is a green tea produced in Xihu and its surrounding areas (Hangzhou, China). As a famous green tea with Protected Designation of Origin (PDO), Longjing is recognized as one of the top green teas for its special appearance (flat and straight leaves), flavor, and taste. Various methods for distinguishing Longjing from other teas have been reported [4–6]. However, little information has been available on the feasibility of discriminating Longjing from its three specific subproducing areas, namely, Xihu, Qiantang, and Yuezhou. As the quality and prices of Longjing tea from the above three producing areas are different, it is necessary to develop effective analysis methods for discrimination of Longjing from different subproducing areas.

Because of the similarity (processing, appearance, and taste) among different subproducing areas, the specific geographical origins of Longjing are usually distinguished by sensory analysis. However, because it is very expensive and may take years to train a tea taster, it would be more efficient to use some nonhuman techniques. Recent years have witnessed increased applications of electronic tongue technology to analysis of wines, milk, tea, beer, juice, and so on [7–10]. In these applications, a very good time stability and sensitivity are obtained using electronic tongue sensors. Moreover, a good correlation between human and electronic tongue judgment has been observed, which makes it a promising alternative to human sensory analysis of teas.

This paper was focused on developing a rapid analysis method for discriminating specific subproducing areas of Longjing tea by electronic tongue and chemometrics. Robust principal component analysis (ROBPCA) [11, 12] was used to detect outliers in each class. Partial least squares discriminant analysis (PLSDA) [13] was used to develop the classification model.

## 2. Experimental and Methods

### 2.1. Tea Samples and Electronic Tongue Analysis

155 Longjing samples were collected from the local tea plantations in Xihu (32 samples, class A), Qiantang (59 samples, class B), and Yuezhou (64 samples, class C). All the samples were preserved in a cool (about 4°C), dark, and dry place with integral packaging before preparation of tea extract. Six gram of each sample was added with 250 mL boiling deionized water and infused for 10 min. The infusion was then filtered into a beaker and cooled to the room temperature (25°C) by water bath for electronic tongue analysis.

The tea infusion was analyzed by an ASTREE Electronic Tongue system (Alpha M.O.S., Toulouse, France). The detection system consists of one reference electrode (Ag/AgCl) and 7 liquid cross selective sensors (ZZ, BA, BB, CA, GA, HA, and JB). The cross-sensitivity and selectivity of the sensor array are listed in Table 1. The sensors array analyzed the solutions of tea samples with sampling interval of 1 s. Each sample was measured for 150 s, which can ensure a stable response.

### 2.2. Data Preprocessing, Outlier Detection, and Data Splitting

All the data analysis was performed on MATLAB 7.0.1 (Mathworks, Sherborn, MA, USA). The responses of the 7 sensors reported at 150 s were used for the subsequent data analysis. Outliers in the data would degrade the classification models and prediction performance, so robust principal component analysis (ROBPCA) was used to detect outliers in each class of samples. ROBPCA can obtain robust projections of the original data points and avoid the masking effects caused by multiple outliers. With the computed robust score distance (SD) and orthogonal distance (OD), ROBPCA can classify an object into one of the four groups: regular points (with small SD and small OD), good PCA-leverage points (with large SD and small OD), orthogonal outliers (with small SD and large OD), and bad PCA-leverage points (with large SD and large OD). With outliers deleted, the DUPLEX algorithm [14] was used to divide the measured data into a training set and test set. DUPLEX algorithm can obtain a test set of samples distributed uniformly in the range of training samples.

### 2.3. PLSDA

For multiclass classification, PLSDA can be performed by regressing each column of a dummy response matrix **Y** on the measured data **X** by PLS. For the *i*th (*i* = 1, 2, and 3) column in **Y**, an element is set a value of 1 if the corresponding object is from class *i*; otherwise, it is assigned a value of 0. For prediction, a new sample is classified into class *i* when the *i*th element of its predicted response vector is nearest to 1. Monte Carlo cross validation (MCCV) [15] was used to estimate the number of components in PLSDA by minimizing the mean percentage error of MCCV (MPEMCCV):

(1) $$\begin{array}{c} \text{MPEMCCV}=\frac{\sum_{i=1}^{B}N_{i}}{\sum_{i=1}^{B}M_{i}}, \end{array}$$

where *B* is the numbers of MCCV data splitting, *M*_*i*_ is the number of prediction objects, and *N*_*i*_ is the number of wrongly predicted for the *i*th MCCV data splitting. Model sensitivity and specificity of prediction for each class were used to evaluate the performance of classification models:

(2) $$\begin{array}{c} \text{Sensitivity}=\frac{\text{TP}}{\text{TP}+\text{FN}},\\ \text{Specificity}=\frac{\text{TN}}{\text{TN}+\text{FP}}, \end{array}$$

where TP, FN, TN, and FP represent the numbers of true positives, false negatives, true negatives, and false positives, respectively. For classification, objects in each class were denoted as positives and the other two classes were denoted as negatives.

## 3. Results and Discussion

**Figure 1:**
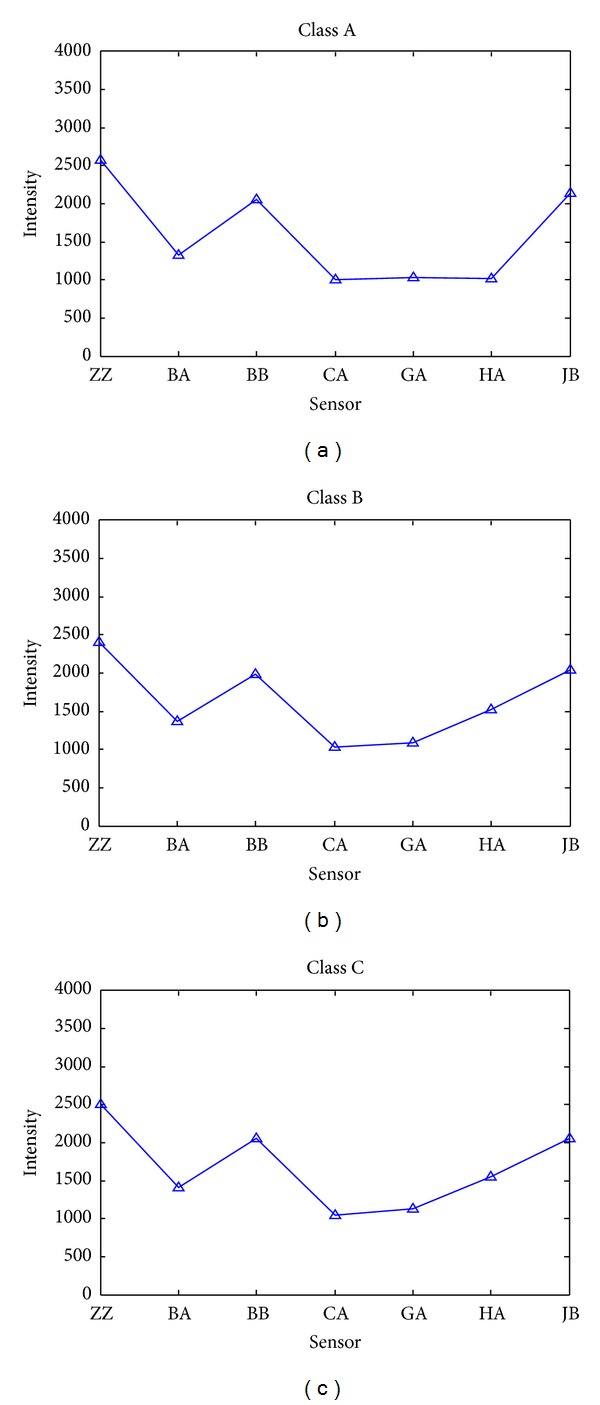
The average feature spectrum of Longjing tea of class A, class B, and class C.

**Figure 2:**
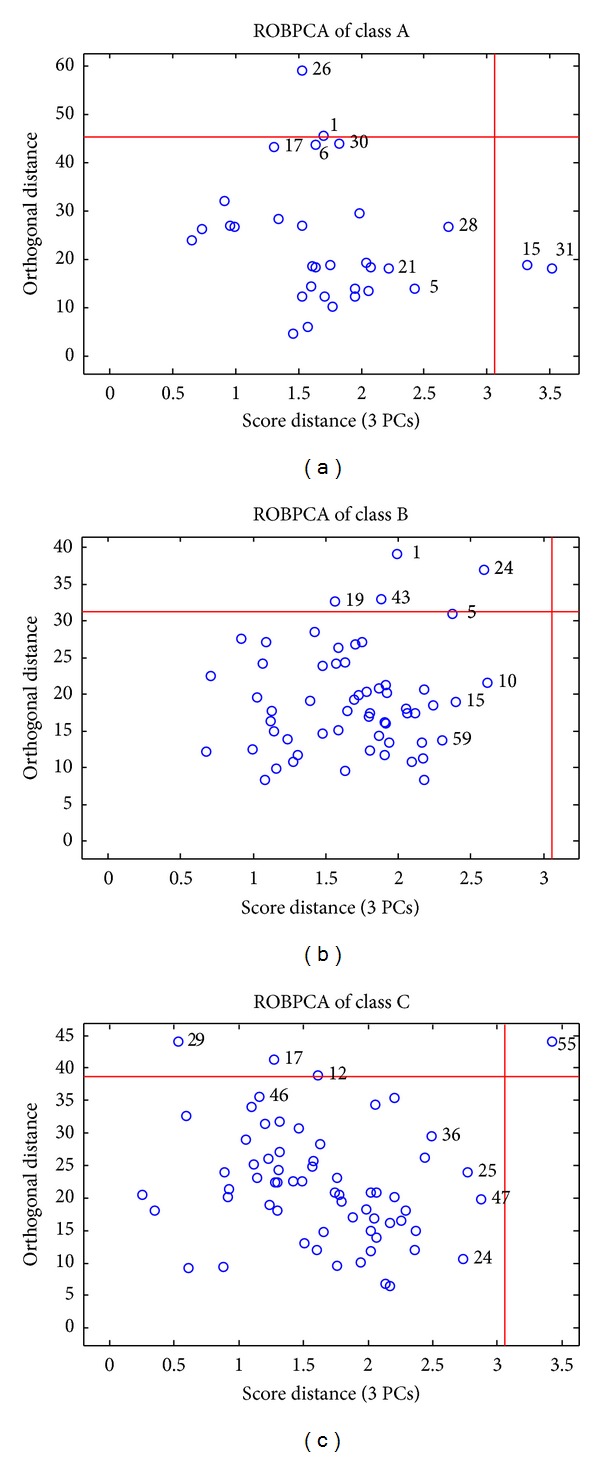
ROBPCA of the electronic tongue data: the outlier plots for Longjing samples from Xihu (class A), Qiantang (class B), and Yuezhou (class C).

The average electronic tongue features for each class of Longjing tea are shown in Figure 1. Seen from Figure 1, the features of the three classes have very similar response patterns. The features of class B and C are very similar and different from those of class A, especially in the responses of GA and HA. ROBPCA was used for outlier detection with a significance level of 0.05. Because each class of tea has a different probability distribution, ROBPCA models were performed separately on each class.  Figure 2 demonstrates the ROBPCA plots for the three classes. For each class, a ROBPCA model with three principal components (PCs) was selected because including more PCs would not reduce the residuals significantly. Three PCs explained 89.7, 91.5 and 92.1 percents of the total variances of each class, respectively. For outlier diagnosis, bad PCA-leverage points, good PCA-leverage points and orthogonal outliers were excluded to obtain a representative data set distributed in the entire range of measured samples. The numbers of objects with large SD and OD were denoted. As a result, for class A, two orthogonal outliers (objects 1 and 26) and two good PCA-leverage points (objects 15 and 31) were deleted; for class B, four orthogonal outliers (objects 1, 19, 24, and 43) were deleted; for class C, three orthogonal outliers (objects 12, 17, and 29) and one bad PCA-leverage point (object 55) were deleted. 

The DUPLEX algorithm was then performed to divide the remaining 143 tea samples into training and test objects. Finally, 95 samples (class A, 18; class B, 37; and class C, 40) were used for training and 48 samples (class A, 10; class B, 18; and class C, 20) for prediction. PLSDA model was developed and MCCV was used to select the number of latent variables (LVs). For MCCV, the original 95 training samples were randomly split into training (50%) and test objects (50%) for 100 times. The lowest MPEMCCV value was obtained with a three-component PLSDA model.

**Figure 3:**
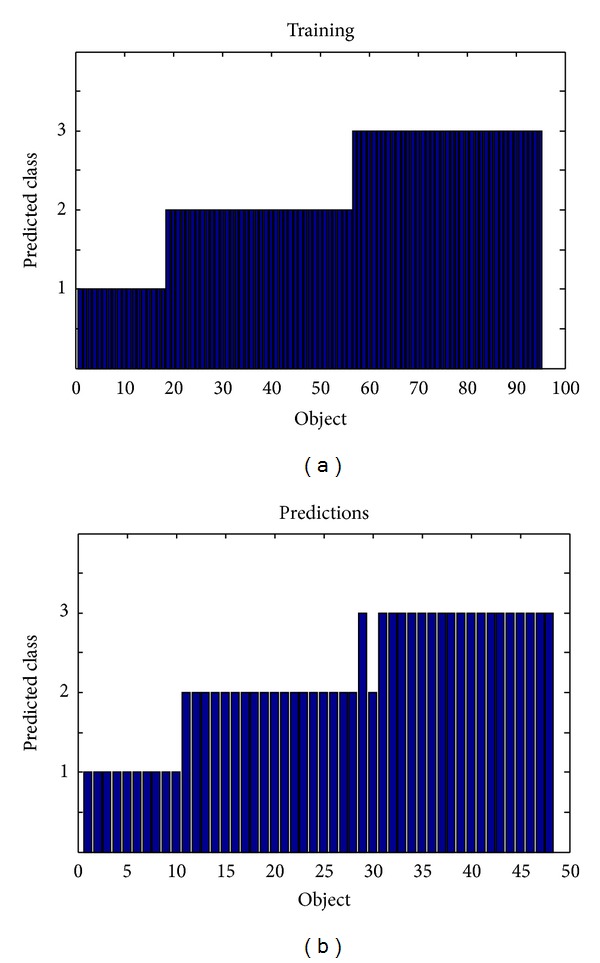
PLSDA training (objects 1–18, class A; objects 19–55, class B; objects and 56–95, class C) and prediction (objects 1–10, class A; objects 11–28, class B; and objects 29–48, class C) of the specific Longjing geographical origins. The height of a bar indicates to which class an object is assigned by PLSDA.

The training and prediction results of a three-component PLSDA are demonstrated in Figure 3. The sensitivity/specificity of PLSDA for classes A, B, and C was 1.000(10/10)/1.000(38/38), 1.000(18/18)/0.967(29/30), and 0.950(19/20)/1.000(28/28), respectively. The training accuracy was 1 and for prediction only one object from class C was wrongly assigned to class B, indicating the effectiveness of electronic tongue for classification of Longjing tea samples.

## 4. Conclusions

Rapid and effective discrimination of Longjing green tea from different subproducing areas was performed using electronic tongue and chemometrics. The sensitivity/specificity of PLSDA for classes A, B, and C was 1.000/1.000, 1.000/0.967, and 0.950/1.000, respectively. Electronic tongue and chemometrics can provide a rapid and reliable tool for discriminating the specific producing areas of Longjing. Compared with human sensory analysis, this method is easier to perform and the more attractive economically. In the future studies, the comparison of chemical methods, for example, LC/UV/MS, to the electronic tongue analysis will be performed to investigate the statistical correlation between the chemistry and the tastes of Longjing tea.

## Figures and Tables

**Table 1:** Sensor sensitivity (pC) of ASTREE electronic tongue system.

Basic tastes	Substances	ZZ	BA	BB	CA	GA	HA	JB
Sour	Citric acid	7	6	7	7	7	6	6
Salty	KCL	7	4	4	5	4	4	4
Sweet	Glucose	7	4	7	7	4	4	4
Bitter	Caffeine	5	4	4	5	4	4	4
Savory	L-arginine	6	4	6	5	5	4	5
